# When a Major Orthopaedic Device Manufacturer Does Not Offer Replacement Components for Revision Surgeries

**DOI:** 10.1016/j.artd.2026.102142

**Published:** 2026-09-22

**Authors:** Brian J. McGrory, Samantha Blau, Melissa Graham, Stephen Tower

**Affiliations:** a Department of Orthopedics, MaineHealth, Portland, ME, USA; b University of New England College of Osteopathic Medicine, Portland, ME, USA; c MaineHealth Institute for Research, Portland, ME, USA; d Anchorage Fracture and Orthopedic Clinic, Anchorage, AK, USA

**Keywords:** Revision total hip arthroplasty, Mechanically assisted crevice corrosion, Expanded access, Orthopaedic device regulation, Femoral head exchange

## Introduction

Put yourself in the place of your patient: undertaking hip or knee arthroplasty for diminution of pain and return of mobility, only to find out a decade later that your implant is corroding and the appropriate replacement part is not commercially available in the United States. For a percentage of patients, this isn’t hypothetical—it’s reality. When artificial joints fail, early or late, surgeons and their patients are sometimes left with no guarantee of appropriate, compatible components. In such cases, the options are less suitable or riskier implant parts, higher surgical complexity involving the removal of otherwise well-positioned and ingrown components, or a lengthy and expensive compassionate use approval process for custom implants (via the Expanded Access pathway). This situation is unacceptable, and manufacturers should be held accountable.

Hip, knee, and shoulder arthroplasty are among the most common surgeries performed globally. Over 1.5 million hip and knee replacements are performed annually in the United States alone, and that number is projected to be 5 million in 2040 [1]. More than 100 companies have made hip and knee replacements in the United States, but the market is currently dominated by 5 major manufacturers [2,3]. These devices often last 25 years or more, but historic failure due to infection, instability, fracture of bone around the implant, prosthetic wear, and implant breakage or corrosion occurs in between 18 and 42 percent over the lifetime of any given patient [4,5]. Contemporary implants may be more durable, but recent revision burden appears to remain consistent between 7 and 10.4 % over time [6].

One of the more common indications for late revision in total hip replacements, for example, has changed in the last 2 decades from the consequences of polyethylene wear to the periprosthetic and systemic complications of taper corrosion. Such cases in our experience are both under-recognized and undertreated. Any hip with a modular cobalt-chromium component (acetabular or femoral) is at some degree of risk [7,8] though some specific implant systems are at high or extreme risk. [9],[7,10,11]. Partial or modular revision is often the most appropriate course of action for these patients, but this depends on the availability of compatible FDA (Food and Drug Administration)-approved replacement implants. If FDA-approved replacement implants are not available, a less favorable surgical option may be required. This may involve some combination of delayed intervention, more invasive surgery, more expensive treatment, and greater risk for the patient.

## Our clinical experience with the DePuy Synthes SROM femoral stem

The SROM stem (DePuy Synthes, Warsaw, IN) has been used in our practices for a select number of primary and revision total hip replacements for over 2 decades. We have used modular stems for specific femoral deformities [12], in addition to revision cases where rotational, offset, and length options were crucial. Neither the American Joint Replacement Registry nor industry reports provide precise, publicly available counts of S-ROM stem implantations since its FDA approval in the mid-1980s, but the stem is described as having “achieved a well-deserved prominence” and “stood the test of time” with widespread use in both primary and revision total hip replacement [11,13,14]. As of this Viewpoint submission, it is still manufactured and sold in the DePuy Synthes portfolio. Furthermore, as one of the most successful revision implants, the SROM stem is extremely well fixed to bone and has an excellent track record vis-à-vis stem aseptic loosening [14,15].

A recently recognized failure mode of total hip arthroplasty (THA) is mechanically assisted crevice corrosion (MACC)—a process of corrosion at the bore and trunnion junction of the femoral component in metal-on-polyethylene or metal-on-metal THA with a cobalt alloy femoral head [9,16,17]. If left untreated, patients experiencing MACC can have worsening pain and disability, irreversible tissue loss, instability, increased infection rate, and in some instances, systemic illness. Although common in certain implants [18,19], it can occur in any modular arthroplasty where a cobalt alloy couple exists. In one of our experiences (B.J.M.) with primary cases, the SROM stem was used in 181 of 7219 (2.5%) hip replacements. Of these 181 implants, 4 (2.2%) have been in hip arthroplasty patients who presented with symptoms and have been subsequently diagnosed with MACC.

If diagnosed early, patients with MACC may be offered a revision surgery that is considered straightforward. Although risks may be higher than for other revisions [20], the morbidity is limited because the femoral head is most often exchanged for a revision ceramic head after debriding and removing the source of cobalt ions, corrosion, and metallic debris [16]; that is, if the replacement part for the corroding femoral head is commercially available. The revision ceramic head in this instance is a product that includes the ceramic head itself and a sleeve of titanium alloy that bypasses the imperfections of the previously failed trunnion, therefore decreasing the risk of ceramic head fracture (Fig. 1) [20,21].

**Figure 1:**
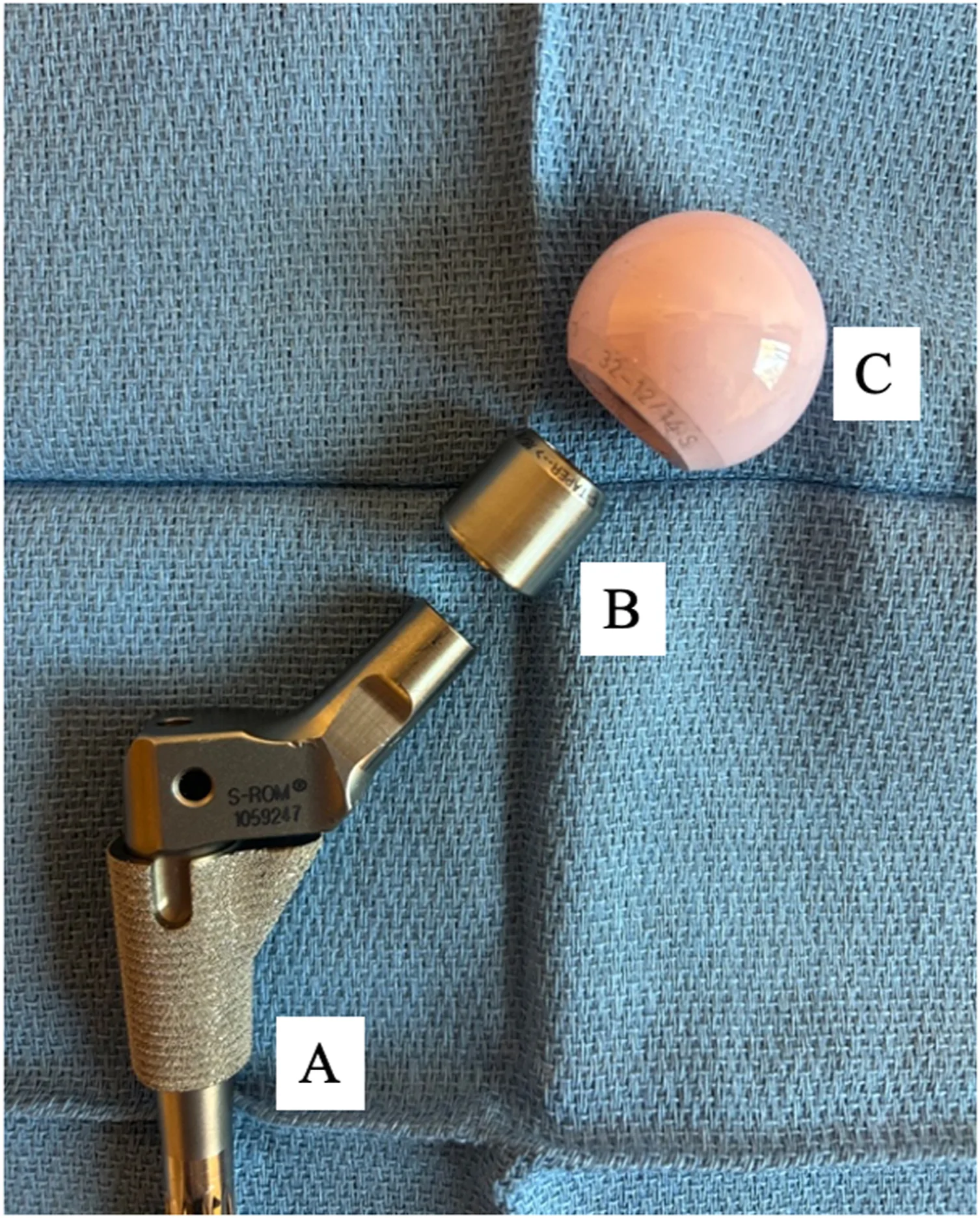
Unassembled modular SROM implant with components needed for successful revision for mechanically assisted crevice corrosion. The parts shown are the (a) femoral component; (b) titanium alloy sleeve when femoral component retained (not available from DePuy Synthes); (c) ceramic femoral head.

In the case of the SROM implant, the manufacturer does not offer this option; notwithstanding, the appropriate replacement revision implant would be easy and relatively inexpensive to manufacture and make available. Despite these known issues, the orthopaedic device manufacturer (ODM), in this case DePuy Synthes, has provided no rationale (financial or otherwise) for why they do not offer this much-needed option to clinicians and their patients.

## Impact and treatment options

Surgeons are bound by the principle of nonmaleficence, which is the responsibility to minimize harm to the individual patient and reach a beneficial outcome. When an ODM does not offer needed revision parts for the implants they manufacture and sell, the patient, treating surgeon, and hospital are left with several less safe, more expensive, and more morbid options.

### Complete stem removal

Stem removal is a drastic response if the trunnion is in satisfactory repair. It is a morbid option and will certainly require a longer, more complex surgery with likely extended trochanteric osteotomy. Other risks associated with the length and complexity of the surgery include intraoperative fracture, femoral bone loss, and cortical perforation. The extensive proximal ingrowth to the sleeve (metaphyseal portion) of the S-ROM (Sivash-Range Of Motion) stem also makes significant bone loss very likely during removal. The rate of intraoperative fractures that occur with complete stem removal is approximately 12%, and the incidence in cementless systems, such as the DePuy S-ROM modular system, rises to 19% [22]. While most intraoperative fractures can be successfully managed and may not typically compromise long-term function [[23], [24], [25], [26]], it is an unnecessary risk for the patient to go through while undergoing an already strenuous revision operation. Furthermore, bone loss [27] and cortical perforation [28], as well as the increase in operating time, add to the already high risk of nonunion and infection in MACC revisions [20].

### Revision with a primary (non-sleeved) ceramic head or metal head

Using a primary ceramic head without a revision sleeve is “off-label,” that is, not specifically authorized by the FDA, for revision cases and increases the risk of ceramic fracture; fracture leaves the surgeon no choice but to use another cobalt alloy femoral head, and likely not addressing the reason for revision [23]. Likewise, using another cobalt alloy metal head in the treatment of MACC is associated with continued corrosion and does not resolve the reason for revision [29].

### Custom implant

Custom implants are possible but expensive and often require FDA and institutional review board (IRB) approvals. Obtaining these approvals is both time- and labor-intensive. This option also requires ODM cooperation, which may not be forthcoming. Lastly, manufacturing errors and limited operative options for soft tissue tensioning and leg length restoration make this approach problematic. Of note, one ODM (Zimmer Biomet) initially offered a custom sleeve to treat corrosion in its legacy taper but subsequently manufactured FDA-approved sleeves for use with ceramic heads [30].

### Use of BioBall international implant

The Merete BioBall modular hip system (Merete GmbH, Berlin, Germany) is an orthopaedic device manufactured in Europe that allows the creation of a new modular junction between a retained femoral stem and a replacement femoral head during revision THA. Although its use is widely reported in the European literature [31], there is no FDA clearance or approval for its clinical use in the United States. Published studies describe its use almost exclusively in European centers and do not reference FDA 510(k) clearance or premarket approval. This distinction is critical, as medical devices manufactured and marketed in Europe require additional regulatory approvals in order to receive authorization from the FDA for use in the United States.

Most currently available orthopaedic devices are approved through the FDA 510(k) premarket notification system rather than through clinical trials [32,33]. Additionally, the use of the BioBall system in the United States does not meet the criteria for the FDA’s Humanitarian Use Device Exemption, which applies only to conditions affecting less than 8000 patients annually [34]. Unfortunately, revision THAs exceed this threshold. In our opinion, these cases represent a predictable and relatively common clinical scenario that supports the need for all companies to make replacement parts for their modular hip systems.

Again, there is a significant burden to clinicians who want to obtain approval for the BioBall system for their patients. Surgeons must work through the Expanded Access pathway in order to obtain regulatory approval to use the BioBall device.

### The compassionate use or Expanded Access option

According to the FDA, “Sometimes called compassionate use, Expanded Access is a potential pathway for a patient with a serious or immediately life-threatening disease or condition to gain access to an investigational medical product (drug, biologic, or medical device) for treatment outside of clinical trials when no comparable or satisfactory alternative therapy options are available” [35].

Obtaining an Expanded Access/compassionate use device requires the commitment of time and resources by the treating clinician and staff. The treatment of a patient with an otherwise unapproved device requires multiple steps, communication within and outside of the hospital organization, as well as obtaining various approvals (regulatory and institutional). Medical necessity must be justified, and an independent assessment of the case must be made after confirming that the patient will qualify for Expanded Access/compassionate use. If appropriate, following review of the specific case, the ODM will provide their authorization for use in advance of the surgeon (and/or device company) seeking out regulatory approvals. Finally, FDA and IRB approvals must be obtained before the patient can be consented and, ultimately, treated. This process is outlined in Figure 2.

**Figure 2:**
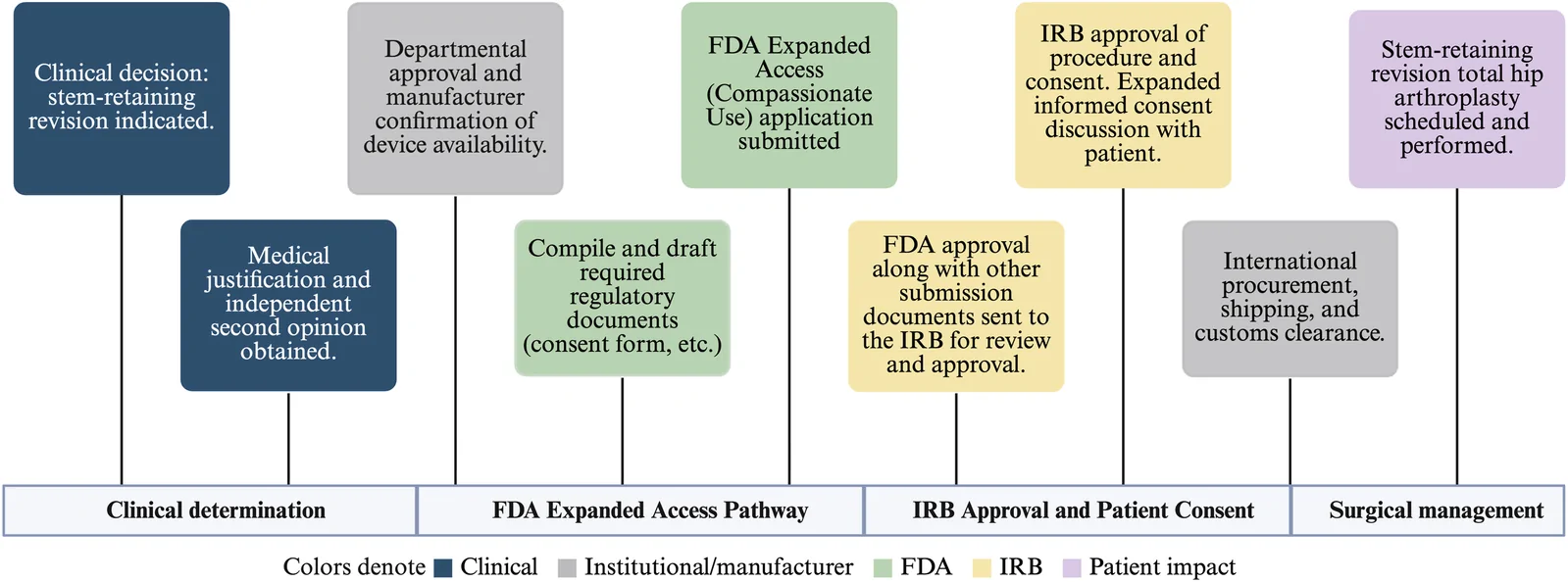
Process and time burden associated with third-party system use in stem-retaining hip revision. Regulatory and institutional processes required to perform stem-retaining revision total hip arthroplasty using a non–FDA-cleared device under the Expanded Access (compassionate use) pathway. Colors denote clinical (blue), institutional/manufacturer (gray), FDA (green), IRB (yellow), and patient-impact (purple) domains.

Made with BioRender.

In addition to routine clinical care, following consent and treatment, there are follow-up, adverse event, and annual regulatory reporting requirements for the FDA and IRB, as well as the device company. In some cases, the device company will be responsible for FDA submissions; however, that is not always the case.

Budget restrictions in many hospitals may deem these surgical revisions financially unsustainable, as the implant alone costs in excess of 20,000 USD. Moreover, Medicare or insurance reimbursement may not reliably cover revision pathways that fall outside of FDA guidelines [36]. Consequently, procedural or device costs may be shifted to patients or institutions. Furthermore, the regulatory process may lead to delays in treatment, referral to alternative centers, or restricted access to clinically appropriate revision options. These barriers may disproportionately affect Medicare and Medicaid beneficiaries [37], particularly low-income patients treated at safety net hospitals, thereby exacerbating socioeconomic disparities in access to complex revision arthroplasty.

## Ethical and policy recommendations

As Pean et al [38]. have pointed out, “*the use of orthopaedic implants intertwines the incentives, interests, and objectives of 3 key stakeholders: (1) patients, (2) surgeons, and (3)* ODMs*.”* In our experience with the SROM femoral implant, a lapse in manufacturer accountability is the prominent concern.

When a manufacturer does not provide a compatible replacement femoral head for a common and extensively implanted product, the burden of weighing various suboptimal options is now shifted to the surgeon and patient. The need for femoral head exchange occurs not only in cases of corrosion but also in routine revision situations where the femoral stem remains fixed and functional. The ODM involved has made what seems to be an unethical and illogical decision not to support their commonly used femoral implant with a safe and appropriate replacement femoral head.

In the absence of revision components made by the manufacturer, surgeons are forced to accept unnecessary risks of femoral head fracture, use a metal head when it should not be used, or take on a significant time and regulatory challenge—often without compensation—in order to offer the patient the best possible outcome. All the while, a seemingly simple and accepted solution is available: manufacture and sell the appropriate replacement components.

The process used to obtain the correct replacement femoral head for one commonly implanted total hip illustrates how ODMs can introduce avoidable complexity into an otherwise routine aspect of revision care; our view is that such barriers are difficult to justify from both clinical and ethical perspectives. As noted above, DePuy Synthes does not offer ceramic femoral head implants for its 11/13 taper, despite the widespread use of this taper in primary and revision THA, thereby limiting straightforward revision options. Because current regulations do not require ODMs to offer appropriate implant parts for revision, the burden on surgeons and ultimately patients persists despite a relatively predictable need for replacement parts over the lifespan of an implant. Therefore, we suggest that upon FDA approval, orthopaedic device companies should be required to manufacture appropriate revision components alongside primary implant systems. This solution would allow surgeons to reduce unnecessary risk and inequity in revision hip arthroplasties and satisfy the principle of nonmaleficence.

## Conclusions

Physicians, including orthopaedic surgeons, are morally obligated to minimize risk and maximize outcomes for their patients. In joint replacement surgery, the process of patient optimization and surgical preparation are paramount. In preparation for revision surgery, the surgeon must do everything in their power to assure an efficient and low-risk surgery. Ultimately, this begins with the appropriate implants to execute the surgical plan. When a major ODM does not offer appropriate replacement components for a revision, this is problematic.

We have outlined above the costly, cumbersome, and work-intensive process used to obtain the correct implant for one commonly implanted femoral stem that can fail by the process of MACC. Our view is that this process, however costly and inconvenient, is the correct path to take, despite the challenges. We also posit that the ODM that has sold the SROM implant, DePuy Synthes, should make available this needed implant for a reasonable price and obtain FDA approval for its use. Similar to the Zimmer Biomet decision to offer revision heads for its legacy 6-degree taper stems, DePuy Synthes should change its policy and offer revision ceramic femoral head implants. Almost all other major ODMs offer analogous parts for their implants, both currently sold products and also legacy products, and this is a correct and ethical approach. Some other ODMs not discussed may also have limited or no options for replacement parts, but the case of the SROM 11/13 taper appears to be the most flagrant example of a common and frequently used implant without appropriate replacement parts being offered. As MACC and adverse local tissue reaction grow in prevalence and are more recognized, the problem will only grow. We therefore recommend that if companies like DePuy Synthes do not offer replacement parts for their products on their own accord, that the business practice of offering needed and compatible modular implants for revision surgery should be mandated by the appropriate regulatory agency.

## Conflicts of interest

B.J. McGrory receives royalties from Innomed, Inc. and Smith & Nephew, Inc.; receives institutional research support from Smith & Nephew, Inc. and Zimmer Biomet, Inc.; and is a member of the AAHKS Publications committee; and Editor-in-Chief emeritus of Arthroplasty Today; all other authors declare no potential conflicts of interest.

For full disclosure statements refer to https://doi.org/10.1016/j.artd.2026.102142.

## CRediT authorship contribution statement

**Brian J. McGrory:** Writing – review & editing, Writing – original draft, Supervision. **Samantha Blau:** Writing – review & editing. **Melissa Graham:** Writing – review & editing. **Stephen Tower:** Writing – review & editing.

## Appendix A Supplementary data

Conflict of Interest Statement for Graham

Conflict of Interest Statement for Blau

Conflict of Interest Statement for Tower

Conflict of Interest Statement for McGrory
