# Supplementary material for: When a Major Orthopaedic Device Manufacturer Does Not Offer Replacement Components for Revision Surgeries
Source: Arthroplast Today. 2026 Sep 22;41:102142. doi: 10.1016/j.artd.2026.102142 (PMC13633937; doi:10.1016/j.artd.2026.102142)
Supplement: Supplementary file 3 [file mmc3.pdf]

CONFLICT OF INTEREST STATEMENT

American Association of Hip and Knee Surgeons

(Adopted from the American Academy of Orthopaedic Surgeons disclosure statement)

The following form **must be filled out completely and submitted by each author (example, 6 authors, 6 forms). All items require a response. If there is no relevant disclosure for a given item, enter "*None.*"**

Manuscript Title **When a Major Orthopaedic Device Manufacturer Does Not Offer Replacement Components for Revision Surgeries**

1. Royalties from a company or supplier (The following conflicts were disclosed) None

2. Speakers bureau/paid presentations for a company or supplier (The following conflicts were disclosed) None

3A. Paid employee for a company or supplier (The following conflicts were disclosed) No

3B. Paid consultant for a company or supplier (The following conflicts were disclosed) No

3C. Unpaid consultants for a company or supplier (The following conflicts were disclosed) No

4. Stock or stock options in a company or supplier (The following conflicts were disclosed) No

5. Research support from a company or supplier as a Principal Investigator (The following conflicts were disclosed) No

6. Other financial or material support from a company or supplier (The following conflicts were disclosed) No

7. Royalties, financial or material support from publishers (The following conflicts were disclosed) No

8. Medical/Orthopaedic publications editorial/governing board (The following conflicts were disclosed) No

9. Board member/committee appointments for a society (The following conflicts were disclosed) No

Each author must sign AND print or type his/her name, date and submit a separate form

In addition, one BLINDED Conflict of Interest form (no author names used) should be submitted per manuscript with all author disclosures.

Stephen Tower | Stephen Tower | 1/13/2026

Author Name (Print or Type) | Author Signature | Date
